# Spleen Size and Function in Sherpa Living High, Sherpa Living Low and Nepalese Lowlanders

**DOI:** 10.3389/fphys.2020.00647

**Published:** 2020-06-30

**Authors:** Pontus Holmström, Eric Mulder, Victor Starfelt, Angelica Lodin-Sundström, Erika Schagatay

**Affiliations:** ^1^Department of Health Sciences, Mid Sweden University, Östersund, Sweden; ^2^Department of Nursing Sciences, Mid Sweden University, Sundsvall, Sweden; ^3^Swedish Winter Sports Research Centre, Mid Sweden University, Östersund, Sweden

**Keywords:** high altitude, Breath-holding, hypobaric hypoxia, adaptation, Altitude Populations, spleen contraction, cardiovascular diving response

## Abstract

High-altitude (HA) natives have evolved some beneficial responses leading to superior work capacity at HA compared to native lowlanders. Our aim was to study two responses potentially protective against hypoxia: the spleen contraction elevating hemoglobin concentration (Hb) and the cardiovascular diving response in Sherpa highlanders, compared to lowlanders. Male participants were recruited from three groups: (1) 21 Sherpa living at HA (SH); (2) seven Sherpa living at low altitude (SL); and (3) ten native Nepalese lowlanders (NL). They performed three apneas spaced by a two-min rest at low altitude (1370 m). Their peripheral oxygen saturation (SpO_2_), heart rate (HR), and spleen volume were measured across the apnea protocol. Spleen volume at rest was 198 ± 56 mL in SH and 159 ± 35 mL in SL (*p* = 0.047). The spleen was larger in Sherpa groups compared to the 129 ± 22 mL in NL (*p* < 0.001 compared to SH; *p* = 0.046 compared to SL). Spleen contraction occurred in all groups during apnea, but it was greater in Sherpa groups compared to NL (*p* < 0.001). HR was lower in Sherpa groups compared to NL both during rest (SL: *p* < 0.001; SH: *p* = 0.003) and during maximal apneas (SL: *p* < 0.001; SH: *p* = 0.06). The apnea-induced HR reduction was 8 ± 8% in SH, 10 ± 4% in SL (NS), and 18 ± 6% in NL (SH: *p* = 0.005; SL: *p* = 0.021 compared to NL). Resting SpO_2_ was similar in all groups. The progressively decreasing baseline spleen size across SH, SL, and NL suggests a role of the spleen at HA and further that both genetic predisposition and environmental exposure determine human spleen size. The similar HR responses of SH and SL suggest that a genetic component is involved in determining the cardiovascular diving response.

## Introduction

High altitude (HA) is an extreme environment posing stress on the human body, due to the hypobaric hypoxia which reduces arterial oxygen saturation (SaO_2_; Grocott et al., 2009). The HA regions of the Tibetan plateau, the Andeas, Eastern Africa, and North America currently inhabit over 140 million indigenous people, who permanently reside at HA (>2500 m; Moore et al., 1998). HA populations have been chronically exposed to this ambient hypobaric hypoxic stress for many generations and, through evolutionary adaptation, developed beneficial physiological defense mechanisms not present in lowlanders (Moore et al., 1998; Beall, 2006; Wu and Kayser, 2006; Gilbert-Kawai et al., 2014). While many are well described, there may still be undetected mechanisms to explore, with an important role in enhancing human performance at HA.

The hypoxia of HA causes decrements in maximal oxygen uptake (V̇O_2__*max*_; West, 1984) leading to reduced work capacity in lowlanders (Fulco et al., 1998; Wehrlin and Hallén, 2006). HA natives generally have superior V̇O_2__*max*_ (Chen et al., 1997; Brutsaert, 2008) and work capacity at HA, compared to lowland populations (Kiyamu et al., 2015), with higher SaO_2_ during submaximal work at HA and a smaller arterial oxygen desaturation with increased intensity compared to lowlanders (Brutsaert et al., 2000). HA natives, particularly the Tibetans who have lower SaO_2_ at HA compared to Ethiopians and Andeans (Beall, 2006), appear to have other means to cope with severe hypoxemia, which would be particularly efficient during bouts of exercise at HA with limited oxygen. One means to enhance the oxygen-carrying capacity is to increase the amount of circulating red blood cells (RBC) and thereby hemoglobin concentration (Hb) and oxygen content. This can be achieved both through the long-term effects of hypoxia on erythropoiesis (Siebenmann et al., 2017) and in the short term by spleen contraction (Schagatay et al., 2001).

The spleen stores a supply of non-circulating erythrocytes that represents a potential defense mechanism against hypoxic stress in various mammalian species (Barcroft and Stephens, 1927; Hurford et al., 1996) including humans (Hurford et al., 1990; Schagatay et al., 2001). Spleen contraction can transiently increase hematocrit (Hct) and Hb levels in the systemic circulation during apnea (Schagatay et al., 2001, 2005, 2007; Bakovic et al., 2003) and other situations involving hypoxia. Research in hypoxia physiology found that the spleen response is also initiated by hypoxic eupnoea (Richardson et al., 2008; Lodin-Sundström and Schagatay, 2010) indicating that splenic contraction could be important also at HA. While hypoxia is identified as the main stimulus initiating spleen contraction, hypercapnia has a contributing role (Richardson et al., 2012). Spleen contraction is also initiated by intense exercise (Sandler et al., 1984; Laub et al., 1993) and is likely also initiated by general sympathetic activation (Frances et al., 2008; Purdy et al., 2018).

In freedivers, a positive association between spleen volume and apneic diving performance was found (Schagatay et al., 2012). The Bajau, a freediving marine hunter–gatherer population in South East Asia, were recently reported to possess larger spleens compared to another native population, not exposed to intermittent hypoxia (Ilardo et al., 2018). In addition, given that non-diving Bajau were also found to have large spleens, this was suggested to be an effect of genetic adaptation (Ilardo et al., 2018). However, other studies suggest that spleen volume and function could in fact be affected by training/exposure and thus be influenced by the environment rather than set by genetics (Engan et al., 2014; Rodríguez-Zamora et al., 2015; Schagatay et al., 2020). Few studies have been conducted concerning spleen function at HA. In a study by Engan et al. (2014), the apnea-induced splenic contraction was enhanced following long-term exposure to hypoxia during an expedition to the summit of Everest (8848 m). In the study by Rodríguez-Zamora et al. (2015), a group of trekkers to less extreme altitudes were found to have obtained both larger spleens and a more powerful splenic contraction on return to low altitude. Resting spleen volume and contraction have also been found to be larger in climbers who summited Mount Everest compared to recreational trekkers (Schagatay et al., 2020). Taken together, these studies suggest that a pronounced spleen contraction could be adaptive at HA, by enhancing the overall oxygen-carrying capacity. Recently, spleen volume was also found to be negatively associated with acute mountain sickness (AMS), whereby non-acclimatized lowlanders at HA with bigger spleens developed less AMS symptoms compared to those with smaller spleens (Holmström et al., 2019). This indicates that individual spleen size affects tolerance to hypobaric hypoxia, just as it affects performance during apneic diving (Schagatay et al., 2012). Thus, these studies suggest that both genetic predisposition and previous exposure to hypobaric hypoxia may affect spleen volume and function. These findings attest to an important role of the spleen at HA, which should be studied further in different populations to investigate what determines human spleen size and function in extreme environments.

Another defense mechanism against hypoxia mainly studied during apneic diving is the cardiovascular diving response, which is initiated acutely by apnea and enhanced by facial cooling (Schutema and Holm, 1988; Schagatay et al., 2007). This response diverts oxygen to the brain and the heart, through peripheral vasoconstriction, and along with bradycardia and decreased cardiac output, oxygen consumption is reduced by up to 25% during apnea (Andersson et al., 2004). The diving response is highly associated with apneic diving performance (Schagatay and Andersson, 1998). Freedivers also have a more efficient oxygen-conserving effect compared to non-divers, with a lower heart rate (HR) (Costalat et al., 2017). The absolute HR during apnea is also associated with apneic diving performance (Schagatay, 2009). Taken together, these studies suggest that not only diving bradycardia, i.e., the HR reduction, but also a lower absolute HR during apnea is important for freediving performance. The individual magnitude of apnea-induced bradycardia was recently found to be positively associated with SpO_2_ during normobaric hypoxia, suggesting that a powerful diving response could reveal mechanisms resulting in higher oxygenation at altitude (Starfelt et al., 2018). In a study by Holmström et al. (2019), the apnea-induced diving bradycardia, i.e., the magnitude of HR reduction, was found to be negatively associated with AMS symptoms and positively associated with SpO_2_ during a trek to the Mount Everest base camp (EBC; 5300 m) in non-acclimatized lowlanders, whereby the authors concluded that the physiological responses to apneic diving and HA are functionally linked. These studies indicate that individuals with a more pronounced apnea-induced HR reduction are more tolerant to HA.

There is no previous research conducted on either the spleen response or the cardiovascular diving response in indigenous HA natives, compared to lowlanders. Therefore, our aim was to investigate these two potential defense responses during apnea in a HA population, the Nepalese Sherpa, which are descendants of nomadic HA Tibetans (Gilbert-Kawai et al., 2014), who have been residing at HA for millennia (Beall, 2006; Moore, 2017). We hypothesized that, while Sherpa living at HA (SH) would be expected to both have genetic adaptations and possess exposure-induced acclimatization to HA, Sherpa who migrated to low altitude (SL) may have lost any exposure-induced mechanisms. Therefore, we studied responses to apnea in both SH and SL groups and compared them with non-acclimatized Nepalese of lowland origin (NL), expected to display neither genetic nor exposure-induced responses associated with hypoxia tolerance.

## Materials and Methods

### Participants

Thirty-eight male participants recruited from three different population categories volunteered for the investigation: (1) 21 SH, who were currently living at HA (>2500 m) and working as trekking or climbing guides. All of them had been repeatedly exposed to altitudes >5500 m, and all but three of them in the last 2 months. (2) Seven SL, who had migrated to and been residing in Kathmandu (1370 m) for a minimum of 2 years, and did not work at HA. They had not been exposed to altitudes >3000 m in the last 2 months, but all of them had visited these elevations in the past. (3) Ten NL who were all born and resided permanently in Kathmandu, only two of which had ever visited elevations >3000 m, but not in the last 2 months. They did not belong to any ethnic population with Tibetan origin, nor did they work in environments, which would expose them to hypoxia. Based on power calculations, samples of approximately 10 participants per group were required. Due to recruitment limitations in the field, where particularly the SL is a small group, only seven SL were included.

None of the 38 participants had any prior experience in apneic diving. After receiving detailed written and oral information about the procedures, using an interpreter where necessary, participants gave their written informed consent. The study protocol had been approved by the Regional Committee for Medical and Health Research Ethics in Umeå, Sweden, and the Nepal Health Research Council (NHRC), in Kathmandu, Nepal, and complied with the Declaration of Helsinki.

### Procedures

Tests were conducted in Kathmandu at 1370 m. The experimental procedure consisted of a series of voluntary apneas, which took approximately 30 min. All participants were asked not to eat, drink, or perform any physical exercise within one hour the experimental session. On arrival, participants’ height (cm) and weight (kg) were collected, after which they were seated and asked about their history of HA exposure. Vital capacity (VC) was recorded in duplicate in the sitting position (Vitalograph Ltd., Compact II, Buckingham England) and the larger volume used for analysis. Blood pressure (Omron M41, Omron Healthcare, Europe) and body core temperature (Microlife AG, 9443, Widnau, Switzerland) were measured in duplicate after 5 min of rest to exclude hypertension (>200/100) or fever (>38 C). Prior to the apnea test, participants were seated for a minimum of 10 min (mean ± SD 12 ± 3 min), after which a two-min countdown for the apnea test was initiated.

### Apnea Protocol

**FIGURE 1:**
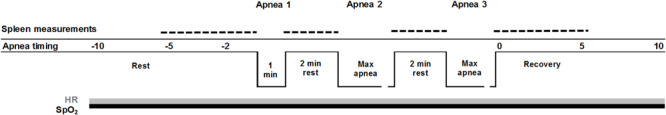
Timing of apneas: apnea 1 (A1), apnea 2 (A2), and apnea 3 (A3), with spleen measurements (dotted line), continuous measures of SpO_2_ (peripheral oxygen saturation, black line), and HR (heart rate, gray line). 15 s of hyperventilation preceded apnea 3 (A3).

Apneas were performed in a sitting position. The protocol consisted in three static apneas (Figure 1), the first of one-min duration (A1) and the following two of maximal voluntary duration (A2–A3), all separated by 2 min of rest (Schagatay et al., 2005; Engan et al., 2014). When 1 min remained prior to each apnea, the participants were notified about the time remaining, when 30 s remained they received a nose-clip and when 10 s remained a second-by-second countdown started. Apneas were preceded by spontaneous breathing and started following a normal expiration and an inspiration on the request to take a deep but not maximal breath, resulting in approximately 85% of VC (Schagatay and Holm, 1996). This is more comfortable during static apneas compared to full lung inflation. The third apnea (A3) was preceded by 15 s of hyperventilation, to reduce arterial tension carbon dioxide (PCO_2_) in order to prolong the apneas. Hyperventilation, involving moderately increased respiratory rate and tidal volume, was demonstrated in advance to the participants. To avoid risk of hypoxic syncope during apnea, if SpO_2_ should fall below 60%, the participants would be told to interrupt the apnea and resume breathing.

### Measurements

**FIGURE 2:**
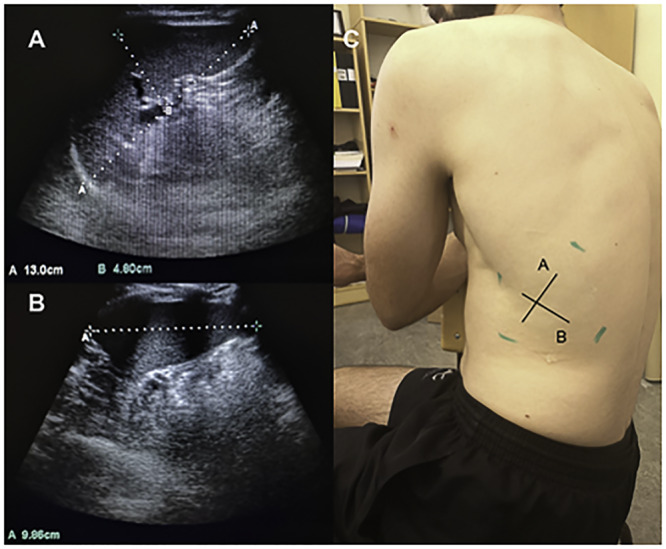
Three axial spleen diameters of maximal length and thickness **(A)** and spleen width **(B)** were collected on the dorsal side of the body **(C)**.

To continuously record HR and SpO_2_, a peripheral finger pulse oximeter (MedAir LifeSense LS1-9R, MedAir AB, Delsbo, Sweden) was used with sensors attached to the index finger. Data were stored via a memory unit (TrendSense, Nonin Medical Inc, MedAir AB, Hudiksvall, Sweden). Spleen diameters were measured via ultrasonic imaging (M-Turbo Ultrasound system, FUJIFILM Sonosite Inc, Bothell, WA, United States; Figures 1, 2A–B). With participants seated, the best individual site for spleen measurement was identified and marked on the left dorsal side of the body (Figure 2C) during approximately 3 min. Spleen images were then taken at this site every minute throughout the protocol until 5 min after the last apnea. For each measurement, two ultrasound images were frozen and the maximal spleen length, width (Figure 2A), and thickness were measured (Figure 2B).

### Analysis

Spleen volume was calculated with the triaxial measurements of length (L), width (W), and thickness (T), using the Pilström equation:

$$V_{s\,p\,l\,e\,e\,n}=(\text{L}\pi \left(\text{WT}-{\text{T}}^{2}\right)/3$$

The formula describes the difference between two ellipsoids divided by two, based on the observed average shape of the spleen (Schagatay et al., 2005; Lodin-Sundström and Schagatay, 2010; Engan et al., 2014). To minimize any influence of the pulsatile changes in spleen volume and minor changes in probe placement, the mean of the two largest consecutive measurement values recorded during the five-min period before the first apnea was used to obtain the individual baseline spleen volume (Schagatay et al., 2012; Holmström et al., 2019). Spleen volume changes that occurred from apneas were calculated by comparing values obtained after apnea and after recovery with baseline volumes.

Baseline values used for calculation of changes of the continuous variables HR and SpO_2_ were obtained as means from the period 90–30 s before the first apnea. The magnitude of the diving response was quantified by the apnea-induced HR reduction (diving bradycardia), expressed as the percentage change between the baseline value and the lowest HR observed during each apnea (maximal HR reduction). The reduction in SpO_2_ was calculated as the percentage change between the baseline value and SpO_2__*nadir*_ ocurring within the 30-s period following each apnea, to account for the circulatory delay from the lung to the finger.

### Statistical Analysis

All data are reported as mean ± SD for the three groups. IBM SPSS statistics (version 25 for windows) was used to run appropriate statistical analysis. Normality distribution of data was tested using Kolmogorov–Smirnov and Shapiro–Wilk tests (*p* > 0.05). Assumptions of homoscedasticity of the data were assessed with Leven’s test of equal variance (*p* > 0.05). To evaluate the within-group variance on apnea-induced spleen volume changes, a one-way repeated-measures ANOVA was conducted with Bonferroni corrections for multiple comparisons. Between-group differences were evaluated with independent samples *t*-test. Meaningfulness of observed effects was estimated by the standardized mean difference [Cohen’s *d*, effect size (ES)] computed as the mean difference divided by the pooled SD. ES were presented along with 95% confidence intervals (CI). An ES of 0.0–0.3 was considered a small effect, 0.4–0.7 a medium effect, and >0.8 a large effect (Lee, 2016). A bivariate correlation analysis with Pearson’s correlation coefficient (*r*) was used to measure the association between selected parametric variables, and Spearman’s rank correlation coefficient (*r*_*s*_) was used on non-parametric variables. Differences were considered significant at *p* < 0.05 and *p* < 0.1 denoted trends.

## Results

The SH were older than the other two groups, but all groups were anthropometrically similar except that VC was higher in SH than in NL (Table 1). Due to technical issues during data collection, SpO_2_ and HR data for seven SH and one SL was excluded from data analysis; thus, these analyses were based on 14 SH and 6 SL.

**TABLE 1:** Anthropometrics of Sherpas living high, Sherpas living low, and Nepalese lowlanders.

	Sherpa high (*n* 21)	Sherpa low (*n* 7)	Nepalese low (*n* 10)	*SH-SL*	*SH-NL*	*SL-NL*
				
				*P*	*P*	*P*
Age (years)	33 ± 9	24 ± 5	23 ± 4	0.006	0.004	0.842
Height (cm)	169 ± 6	172 ± 4	166 ± 4	0.187	0.268	0.052
Weight (kg)	70 ± 9	70 ± 16	65 ± 6	0.913	0.063	0.525
Body mass index (kg × m^–2^)	24.7 ± 2.9	23.6 ± 5.1	23.7 ± 2.6	0.658	0.404	0.981
Vital capacity (VC; L)	4.5 ± 0.8	3.9 ± 0.8	3.9 ± 0.4	0.121	0.035	0.965
VC (L)/height (cm)	2.7 ± 0.5	2.3 ± 0.45	2.3 ± 0.2	0.175	0.045	0.710

*Data are presented as mean ± SD.*

### Spleen Volume During Rest

**FIGURE 3:**
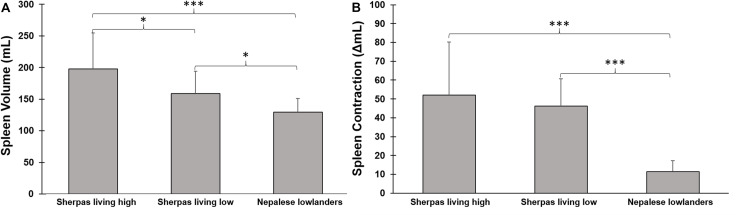
**(A)** Mean (SD) baseline spleen volume during rest in three groups: Sherpas living high (*n* = 21), Sherpas living low (*n* = 7), and Nepalese lowlanders (*n* = 10). ***indicates *p* < 0.001 between groups, and *indicates *p* < 0.05 between groups. **(B)** Mean (SD) spleen volume change after apnea (contraction Δ mL) in three groups: Sherpas living high (*n* = 21), Sherpas living low (*n* = 7), and Nepalese lowlanders (*n* = 10). ***indicates *p* < 0.001 between groups.

Baseline spleen volume was 198 ± 56 mL in SH and 159 ± 35 in SL [*p* = 0.047, ES = 0.75 (0.15–1.61); Figure 3A]. Baseline spleen volume was 129 ± 22 mL in NL, which was smaller than both Sherpa groups [*p* < 0.001 compared to SH, ES = 1.43 (0.57–2.3); *p* = 0.046 compared to SL, and ES = 1.07 (0.0–2.0) Figure 3A]. A large individual variation in spleen volume was seen in all groups.

### Apnea-Induced Spleen Contraction

**FIGURE 4:**
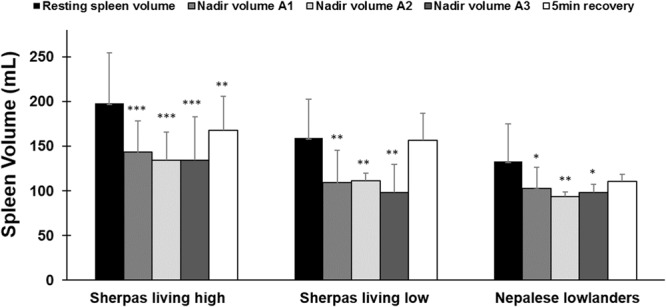
Mean (SD) spleen volume after each apnea compared to resting (baseline) volume and after 5 min of recovery for each group: Sherpas living high (*n* = 21), Sherpas living low (*n* = 7), and Nepalese lowlanders (*n* = 10). ***indicates *p* < 0.001 from baseline. **indicates *p* < 0.01 from baseline. *indicates *p* < 0.05 from baseline.

Spleen volume was reduced in all three groups after apneas (*p* < 0.05; Figures 3, 4). When pooled for all apneas, the spleen volume reduction was similar between SH at 52 ± 28 mL (27 ± 11%), and SL at 46 ± 14 mL [28 ± 7%; *p* = 0.49, ES = 0.24(-0.62–1.09)] but it was greater in Sherpa groups compared to the 11 ± 6 mL (15 ± 5%) reduction found in NL [*p* < 0.001 compared to SH, ES = 1.74, (0.83–2.55); *p* < 0.001 compared to SL, ES = 3.47(1.82–4.76); Figure 3B]. The spleen volume reduction after apnea was positively correlated with baseline spleen volume across all groups (*r*_*s*_ = 0.814; *p* < 0.001).

Within all groups, similar spleen volumes resulted after each of the three apneas (*p* < 0.05; Figure 4); thus, there was no increase in the response across the exposures. 5 min after A3, the spleen volume had increased in all groups. It returned to baseline for SL and NL, but it had increased to a lesser extent for SH (Figure 4). Spleen volume recovery after the apnea series occurred faster and to a greater extent for the Sherpa groups (SH: 4.4 ± 7 mL/min; SL: 10.0 ± 4 mL/min) compared to NL (0.8 ± 2 mL/min; *p* = 0.037 compared to SH; *p* < 0.001 compared to SL).

### Apnea-Induced Diving Response

**FIGURE 5:**
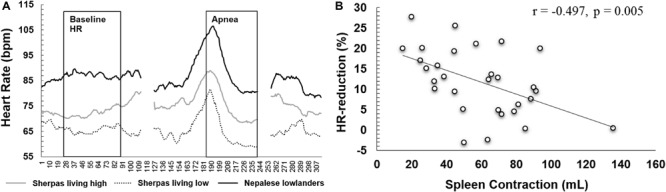
**(A)** Mean heart rate across the first 55 s of apnea 3 for Sherpas living high (gray line, *n* = 13), Sherpas living low (dotted line, *n* = 6), and Nepalese lowlanders (black line, *n* = 9). As individual maximal apnea durations varied, durations exceeding 55 s were cropped, leaving a gap after the apnea followed by the recovery. **(B)** Correlation plot of the individual HR reduction for maximal apneas (mean for A2, A3) and maximal spleen contraction induced by apnea (mL).

Resting HR was lower in Sherpa groups compared to NL (Table 2). HR during maximal apnea was significantly higher in NL compared to SL and tended to be higher compared to SH (Table 2 and Figure 5A).

**TABLE 2:** Functional measures in Sherpas living high and Sherpas living low compared to Nepalese lowlanders.

	Sherpa high (*n* 21)	Sherpa low (*n* 7)	Nepalese low (*n* 10)	*SH compared to SL*			*SH compared to NL*			*SL compared to NL*		
				*P*	ES (*d*)	95% CI	*P*	ES (*d*)	95% CI	*P*	ES (*d*)	95% CI
Apnea duration A1 (s)	48 ± 17	50 ± 15	50 ± 10	0.712	0.1	−1.0–0.71	0.716	0.1	−0.87–0.64	0.913	0.1	−0.9–1.0
Apnea duration A2 (s)	60 ± 25	56 ± 18	53 ± 13	0.645	0.1	0.68–1.03	0.263	0.3	−0.41–1.11	0.640	0.2	−0.7–1.2
Apnea duration A3 (s)	71 ± 27	81 ± 13	65 ± 15	0.194	0.4	−1.26–0.46	0.448	0.2	−0.52–0.99	0.035	1.1	0.0–2.1
HR-reduction A1 (%)	7 ± 9	6 ± 11	16 ± 7	0.954	0.0	−0.9–1.0	0.012	1.1	0.2–2.0	0.066	1.1	0–2.1
HR-reduction A2 (%)	9 ± 7	10 ± 6	20 ± 8	0.791	0.1	−0.8–1.1	0.002	1.5	0.6–2.4	0.013	1.4	0.2–2.5
HR-reduction A3 (%)	8 ± 10	11 ± 5	16 ± 6	0.172	0.4	−0.6–1.3	0.022	1.0	0.1–1.8	0.135	0.9	0.2–1.9
HR_*nadir*_ A1 (bpm)	67 ± 12	62 ± 9	73 ± 5	0.471	0.5	−0.5–1.4	0.085	0.6	0.3–1.4	0.047	1.6	0.4–2.7
HR_*nadir*_ A2 (bpm)	65 ± 12	60 ± 9	69 ± 6	0.410	0.4	−0.5–1.4	0.277	0.4	−0.4–1.2	0.064	1.3	0.1–2.3
HR_*nadir*_ A3 (bpm)	65 ± 12	58 ± 4	72 ± 5	0.217	0.6	−0.4–1.6	0.060	0.8	0.1–1.6	<0.001	3.0	1.4–4.3
Resting SpO_2_ (%) 1400 m	96.8 ± 1.4	98.1 ± 1.2	97.1 ± 0.8	0.097	0.9	−0.07–1.92	0.522	0.25	−0.57–1.06	0.174	1.0	−0.1–2.0
Resting HR (bpm) 1400 m	71 ± 14	66 ± 6	87 ± 7	0.274	0.4	−0.5–1.4	0.003	1.3	0.4–2.2	<0.001	3.5	1.8–4.8

*Data are presented as mean ± SD. All subjects were not able to complete 60 s during apnea 1: 13 in SH, 3 in SL, and 6 in NL. Values for HR reduction, HR_*nadir*_, SpO_2_ at 1400 m, and HR at 1400 m are based on n14 for SH and n6 for SL.*

The relative HR reductions were similar between Sherpa groups (6–11%, NS), but greater in NL (15–20%, *p* < 0.05; Table 2). There was a large individual variation within each group (SH: -27 to 5%; SL: -17 to 15%; and NL: -29 to 4%). The mean HR reduction for maximal apneas was 8 ± 8% for SH, 10 ± 4% [*p* = 0.689, ES = 0.3(-0.7–1.2)] for SL and 18 ± 6% for NL [compared to SH: *p* = 0.005, ES = 1.4(0.4–2.2); compared to SL: *p* = 0.021, ES = 1.5(0.3–2.5)].

Mean HR reduction for maximal apneas was negatively associated with both maximal spleen volume contraction (*r* = -0.497, *p* = 0.005; Figure 5B) and resting spleen volume (*r* = -0.430, *p* = 0.018).

### Apnea Duration and Arterial Oxygen Desaturation

**FIGURE 6:**
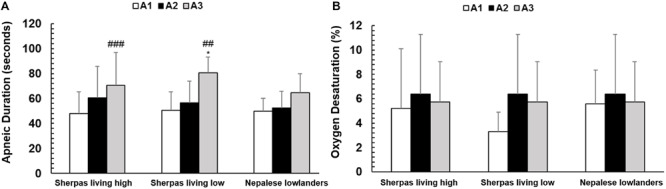
**(A)** Mean apnea duration (seconds) for each of the three groups: Sherpas living high (*n* = 21), Sherpas living low (*n* = 7), and Nepalese lowlanders (*n* = 10). *indicates *p* < 0.05 and ***indicates *p* < 0.001 between groups. **(B)** Peripheral oxygen desaturation during each apnea for each group: Sherpas living high (*n* = 14), Sherpas living low (*n* = 6), and Nepalese lowlanders (*n* = 10).

Apnea durations were similar between Sherpa groups, but maximal apnea duration was longer for SL compared to NL (Figure 6A and Table 2). Resting SpO_2_ was similar between groups (Table 2). The apnea-induced peripheral oxygen desaturation was also similar in all groups (NS; Figure 6B).

## Discussion

This is the first study investigating spleen size and function, and the cardiovascular diving response during apnea in a HA population compared to lowlanders. The principal findings were that (I) resting spleen volume was larger in Sherpa groups compared to NL, (II) SH exhibited larger spleens during rest compared to SL, (III) spleen contraction was similar between Sherpa groups but greater in Sherpa compared to NL, and (IV) the apnea-induced HR reduction, characterizing the diving response, was similar between the Sherpa groups, but of a greater magnitude in NL.

### Spleen Volume and Apnea-Induced Contraction

Indigenous HA populations have evolved an array of beneficial characteristics to cope with the environmentally induced hypoxia at HA (Beall, 2006; Wu and Kayser, 2006; Gilbert-Kawai et al., 2014), and our results suggest that enhanced spleen volume and function is yet another example of such adaptations. We suggest that the spleen functions to fine-tune circulating Hb between the demands on oxygen delivery and to keep viscosity at an acceptable level to limit cardiovascular stress are important at HA, just as other studies have found the same responses important in apneic diving (Schagatay et al., 2001, 2005, 2012; Bakovic et al., 2003).

### Spleens and Genes

The greater spleen volume in Sherpa groups compared to NL is consistent with observations that the Indonesian Bajau population of long-term freedivers had greater resting spleen volume compared to the Saluan, a non-diving population from the same region (Ilardo et al., 2018). Sherpa and Bajau may have similar benefits from the ability to adjust Hb via spleen contraction to cope with hypoxia. However, Ilardo et al. (2018) also reported that non-diving Bajau had equally large spleens as their active divers and concluded that the difference was not attributable to any extent of phenotypic plasticity but had a genetic basis, and they pointed out possible genes responsible for these qualities. However, the progressively falling spleen size across SH, SL, and NL in our study conversely suggests that both genetic predisposition and environmental exposure determine human spleen size. The novel finding that the Sherpa population also has a big spleen would make it interesting to investigate whether the same genetic component is present as in the Bajau. The three groups we studied were anthropometrically similar; thus, differential spleen size between Sherpa residing high vs. low is likely an effect of environmental exposure, while the difference between SL and NL groups is likely reflective of genetic differences. The SH with larger spleens were ∼10 years older than the two other groups, but as all groups were young, we do not expect this to cause any differences, and age and spleen volume are generally not associated (Prassopoulos et al., 1997). The magnitude of the spleen contraction in both Sherpas living high and those living at low altitude is similar to values of elite HA climbers, with about 29% (Schagatay et al., 2020).

Our results did not indicate an exposure-induced enhancement of spleen contraction in the SH as found by Engan et al. (2014) in lowlanders. They reported that the spleen contraction increased after a successful six-week climb to the summit of Mount Everest, with 35 days spent over 5300 m, while baseline spleen volume remained unchanged (Engan et al., 2014). In a follow-up study, we found an increased spleen contraction after a six-week trek to 5300 m but that also resting spleen volume had increased following the trek (Rodríguez-Zamora et al., 2015). The discrepancy in these findings may relate to the difference in HA exposure, where summiting Mount Everest involved exposure to over 8000 m in the “death-zone,” known to result in general catabolism and physiological deterioration, which would most likely also make spleen growth impossible. In line with this, it was recently reported that a group of Mount Everest climbers had bigger spleens and more pronounced spleen contraction compared to less experienced mountaineers (Schagatay et al., 2020). While that study suggested a role of the spleen at HA, it could not be determined if the climbers pre-expedition preparations, which included an average of 6390 m pre-climbing HA exposure, could have increased their spleen volume and function or genetic factors leading to large contractile spleens made them successful climbers (Schagatay et al., 2020). The present study shows that both individual spleen volume and magnitude of spleen contraction during hypoxia are most likely a result of a combination of genetic predisposition and exposure to hypoxia.

A possible long-term acclimatization of the spleen has also been observed in patients with chronic obstructive pulmonary disease (COPD), whereby patients with SaO_2_ < 90% had larger spleens and also a more pronounced contraction after exercise compared to patients with >90% SaO_2_ (Schagatay et al., 2015). Greater resting spleen volume was recently also found following 8 weeks of long-term apnea training (Bouten et al., 2019), while no effect was found after 2 weeks of apnea training (Engan et al., 2013), suggesting that a fairly strong exposure/training dose is required to induce changes. The difference in duration and the severity of hypoxic stress with the abovementioned HA field expeditions (Engan et al., 2014; Rodríguez-Zamora et al., 2015; Schagatay et al., 2020), wherein SaO_2_ is approximately 70% at the summit of Mount Everest (West, 2000), and 80% at EBC (Grocott et al., 2009), also indicate a dose-response relationship. These studies, in accord with the current study, support the view of an environmental influence on spleen volume, rather than only genetic pre-disposition, a response that is also evident in lowlanders. However, the similar spleen contraction between SH and SL may indicate that some adaptation persists as a genetic predisposition to hypoxia tolerance in lowland Sherpa. Despite this, we found an overall association between resting spleen volume and spleen contraction, which has also been reported previously (Holmström et al., 2019).

In summary, the greater spleen volume and contraction observed in the Sherpa groups compared to NL, together with the referred studies, suggests that the spleen plays an important role in optimizing oxygen transport during various situations involving hypoxia, including HA. While the response is enhanced in different groups, which have genetically adapted to hypoxic stress across generations, it is to some extent plastic in response to exposure or training.

Indigenous HA populations have superior work capacity at HA compared to lowland populations (Kiyamu et al., 2015). Although the literature suggests that Tibetans display lower SaO_2_ at HA and approximately 10% reductions in arterial oxygen content, compared to other HA populations (Beall, 2006), they have other superior means to cope with hypoxia during exercise. We believe that the spleen’s contractile function demonstrated in the current study may have a contributing role in enhancing the exercise performance at HA in the Sherpa. This could be by an acute splenic contraction, thus transiently increasing Hb during bouts of exercise, which is consistent with their smaller oxygen desaturation during exercise. The spleen could also have an important role during rest in decreasing blood viscosity by absorbing circulating Hb thereby limiting cardiovascular stress. Spleen volume in other HA populations awaits further studies, to reveal if this is a general feature of HA adaptation, or characteristic of those with Tibetan origin.

We also found that the apnea-induced bradycardia was less pronounced in both Sherpa groups compared to NL, a novel finding which may seem contradictory to the superior hypoxia tolerance and work capacity with limited oxygen in Sherpa. Freedivers, who are also regularly exposed to hypoxia, have a more powerful cardiovascular diving response compared to non-divers, resulting in extended apnea durations (Schagatay and Andersson, 1998). However, the absolute HR was also lower in Sherpa than in NL across the test, both during rest and apnea, which could reflect a lower metabolic rate and a higher parasympathetic activity. The absolute HR during apnea has also been found to be inversely correlated with diving performance (Schagatay, 2009).

The cardiovascular diving response, which conserves oxygen by prioritizing blood flow to hypoxia-sensitive organs such as the brain and the heart, and by bradycardia also reduces myocardial oxygen consumption (Gooden, 1994), preserves arterial oxygen supplies, and prolongs apnea duration (Andersson et al., 2002, 2004). It could therefore be speculated that HA dwellers could benefit from a similar cardiovascular priority system, temporarily directing blood flow to vulnerable tissues during severe hypoxic stress (e.g., during work at HA). In line with this, it was recently found that the diving response, initiated by apnea at low altitude, was negatively associated with AMS symptoms and maintained SpO_2_ at HA in non-acclimatized lowlanders (Holmström et al., 2019). Why then would the HA-tolerant Sherpa have a less developed diving response than lowlanders?

The apnea-induced HR reductions as well as the absolute HR during apnea of the NL in the present investigation are similar to values of native lowlanders reported in several previous studies (Schagatay et al., 2005; Holmström et al., 2019). We speculate that a cause of the Sherpas response to be less pronounced than in the NL could be differences in autonomic regulation of the heart. HA natives exhibit higher parasympathetic activation both at rest and during exercise compared lowlanders, a condition which also remains after migration to low altitude (Zhuang et al., 1993, 2002). The increased vagal tone of HA residents leads to reduced resting HR both at sea level and at HA compared to lowlanders. As the bradycardia stems mainly from increased vagal parasympathetic activity (Gooden, 1994), this may explain why both Sherpa groups developed less pronounced HR reduction during apnea compared to the NL; with high parasympathetic activity even in a resting state, the Sherpa may have limited potential of further lowering of the HR. This may indicate that HA natives benefit less from this cardiovascular priority system compared to native lowlanders, e.g., when going to HA (Holmström et al., 2019).

Another suggested explanation for Sherpa groups exhibiting lower HR both at rest and during apnea compared to the NL could be that this reflects other hypoxia defence mechanisms in Sherpa, due to e.g., a more efficient oxygen distribution compared to non-acclimatized lowlanders (Moore, 2017). This is reflected by the lower cardiac output during maximal exercise when exposed to hypoxia at HA (Moore, 2017) and may be attributed to a more efficient oxygen utilization at the tissue level (Horscroft et al., 2017).

Holmström et al. (2019) found that a large spleen was associated with fewer symptoms of AMS, in line with the present investigation. We also found an inverse relationship between the magnitude of spleen contraction and the extent of diving bradycardia. It could be speculated that the greater vagal tone observed in Sherpa, which leads to less pronounced apnea-induced bradycardia, has motivated the development of a more powerful splenic contraction compared to native lowlanders, to cope with hypoxia. It should be noted, however, that our study was conducted in resting conditions only, and different responses between groups could possibly result during work.

Our observation that SH and SL exhibit similar degrees of HR responses during apnea may indicate that a genetic component is involved in determining the cardiovascular diving response. This is consistent with previous studies, investigating HR responses during apnea in animals (Fahlman et al., 2011), and suggested to be the case also in humans (Ilardo et al., 2018). However, research in freedivers clearly shows that there are also trainable properties of the cardiovascular diving response, leading to a more powerful response with an earlier onset after only 2 weeks of apnea training (Schagatay et al., 2000; Engan et al., 2013). It would be interesting to study the cardiovascular diving response during HA acclimatization in lowlanders.

### Methodological Considerations

The current study design is associated with a number of limitations, which should be acknowledged. A limitation may be that apnea was used in our study to initiate transient hypoxia, which has been shown to initiate spleen contraction (Schagatay et al., 2005), as does short-term normobaric eupneic hypoxia (Richardson et al., 2008), while hypobaric, more chronic hypoxia could possibly generate other effects on the spleen. Apnea also causes peripheral vasoconstriction, which may influence the data output of SpO_2_ and cause misinterpretations (Luks and Swenson, 2011). However, we used a pulse oximeter with a light signal indicating when blood flow was sufficient, and only included valid data. The experimental procedure using apnea and establishing a well-functioning field laboratory allowed physiological testing with standardized conditions for data collection for the included variables. However, the methodology had to be limited to transportable equipment, which could easily be handled in the field, and the focus was therefore limited to a few variables. It would have been valuable to do repeated blood sampling for Hb, in parallel with spleen measurements, but such sampling and analysis was not possible for logistical reasons. We believe, however, that other studies conducted in permanent research laboratories have reliably confirmed that transient spleen contraction is closely associated with transiently elevated Hb (Schagatay et al., 2005, 2015). We also did not take any biological samples to study genetics in this initial study, and our results may therefore be regarded as indirect evidence of genetic predisposition in the Sherpa. The SL group only consisted of seven participants, and six for some of the data, which is a limiting factor of this investigation, although the statistical analysis shows the sample was likely sufficient for the conclusions to be made.

## Conclusion

The observation of the largest spleens in Sherpa living high suggests that spleen size is important at HA. The progressively decreasing spleen size across SH, Sherpa living at low altitude, and native Nepalese lowlanders (NL) suggests that both genetic predisposition and environmental exposure determine human spleen size, which contrasts to the “genes only” conclusions made by Ilardo et al. (2018) concerning spleen size in apneic divers. The similar and superior magnitude of spleen contraction in both Sherpa groups, compared to lowlanders, further suggests that the spleen function to enhance circulating Hb may be another beneficial defense characteristic of HA natives, likely evolved to cope with severe hypoxia. The similar but attenuated HR responses during apnea in Sherpa groups compared to lowlanders suggests that a genetic component is involved; the lower initial HR in both Sherpa groups likely reflects higher parasympathetic activity, which may not allow as much further lowering of HR as in the lowlanders during apnea.

## Data Availability Statement

The datasets generated for this study are available on request to the corresponding author.

## Ethics Statement

The studies involving human participants were reviewed and approved by Regional committee for Medical and Health Research Ethics in Umeå, Sweden and the Nepal Health Research Council in Kathmandu, Nepal. All participants provided written informed consent for participation.

## Author Contributions

PH contributed to the input on study design, planning & organization of laboratory and field study tests and procedures, data collection, data analysis, and manuscript writing. EM contributed to the planning & organization of laboratory and field study tests and procedures, data collection, and proofreading. VS contributed to the organization of laboratory, data collection, and proofreading. AL-S contributed to the organization of laboratory, data collection, and proofreading. ES contributed to the original idea, planning & organization of laboratory and field study tests and procedures, data collection, manuscript writing, and proofreading. All authors contributed to the article and approved the submitted version.

## Conflict of Interest

The authors declare that the research was conducted in the absence of any commercial or financial relationships that could be construed as a potential conflict of interest.
